# Impact of Light Spectrum on Tadpole Physiology and Gut Microbiota in the Dybowski’s Frog (*Rana dybowskii*)

**DOI:** 10.3390/ani15142066

**Published:** 2025-07-13

**Authors:** Haoyu Ji, Baolong Shan, Nan Hu, Mingchao Zhang, Yingdong Li

**Affiliations:** College of Animal Science and Veterinary Medicine, Shenyang Agricultural University, Shenyang 110866, China

**Keywords:** *Rana dybowskii*, light spectrum, gut microbiota, amphibians, metamorphosis, hormonal profiles, 16S rRNA sequencing

## Abstract

*Rana dybowskii*, widely distributed and extensively farmed in northeast China, holds significant economic value, particularly for its fallopian tubes, which are used as a traditional Chinese medicinal tonic known as “Oviductus Ranae.” As the light spectrum is a cost-effective regulatory factor in aquaculture, understanding its effects on the tadpole stage of *R. dybowskii* is critical for optimizing cultivation practices. This study investigated the effects of five light colors (white, red, yellow, blue, and green) on steroid hormone levels and gut microbiota composition in *R. dybowskii* tadpoles. Steroid hormone levels were measured on days 15, 30, 45, and 60 using high-performance liquid chromatography (HPLC), while gut microbial communities were analyzed through high-throughput 16S rRNA sequencing. Results showed that the testosterone (T) level of frogs in green light (group G) peaked on day 60 (2.62 ± 3.70 ng/g). The estradiol (E2) level in blue light (group B) also peaked on day 60 (2.87 ± 0.71 ng/g). Importantly, sex ratio analysis revealed that the proportion of females was highest under blue light, reaching 61.11%. Meanwhile, the richness and diversity of the gut bacterial community of the tadpoles was highest under yellow light, followed by blue light. These data suggest that hormone levels fluctuated and the composition of the gut flora of *R. dybowskii* changed under different light colors. Our results advance *R. dybowskii* physiological knowledge and support aquaculture practices.

## Simple Summary

Light spectrum has a significant effect on the growth and health of aquaculture animals. In this study, we investigated how different light colors (white, red, yellow, blue, and green) affect the growth, sex ratio, hormone levels, and gut bacteria of the tadpoles of *Rana dybowskii*. The results indicate that green light increased testosterone levels, while blue light increased estradiol levels and female rate. The number of intestinal bacteria in yellow light was the highest. These results suggest that light color can affect many physiological parameters of tadpoles, which may help farmers optimize light conditions to improve tadpole health and growth in aquaculture systems.

## 1. Introduction

The light environment (such as photoperiod, light intensity, and light spectrum) plays a key role in many biological processes in amphibians [1]. The spectral variation can profoundly affect amphibians, particularly frogs, which exhibit complex visual systems—most possessing trichromatic or even tetrachromatic vision with sensitivity into the ultraviolet range [2]. For example, the growth of *Pelophylax ridibundus* larvae is accelerated and metamorphosis is shortened under blue light, whereas red light is detrimental to their growth and development [3]. Tadpoles of *Rana cyanophlyctis* exposed to red light were observed to undergo metamorphosis earlier than those reared under white or other light colors [4]. Additionally, artificial light with narrow spectral ranges (e.g., low-pressure sodium lights) may obscure visual cues vital for behaviors like mate selection, especially if those cues rely on color-based sexual dimorphism [5]. The disruption of natural spectral information by artificial lighting may therefore alter phototactic responses, habitat use, and reproductive behaviors in frogs, underscoring the urgent need for field-based studies on spectral and intensity-dependent visual ecology in frogs.

In amphibians, sex steroid hormones such as estrogens, progesterone, and androgens perform multiple physiological functions, including regulating reproductive behaviors, promoting gonadal development, and modulating secondary sexual characteristics [6,7]. Among them, amphibians rely on cortisol (COR) as their primary glucocorticoid, and stress hormones such as cortisol affect the timing and size of amphibian metamorphosis and may affect phenotypic expression later in life [8]. Cortisol secreted by the interadrenal tissue can cause amphibians to adapt to environmental changes, such as environmental deterioration, and can promote the survival of the larvae, but it may also reduce the size of the tadpole metamorphosis [9]. Testosterone is important for the development and seasonal variation in amphibian dimorphic organs [10]. These hormones are influenced by various environmental factors; for instance, temperature can alter sex ratios by affecting steroidogenic enzyme activity in some amphibians [11], and chemical pollutants such as atrazine have been shown to disrupt endocrine function and feminize male frogs [12]. However, studies on the effects of the light spectrum on hormone regulation in amphibians remain limited.

The gut microbiome is closely linked to the host and influences the overall performance, health, and physiology of individual aquatic organisms and species [13]. In addition, factors such as environment, life stage, and life history influence the structure and function of the gut microbiome. For example, hibernation significantly lowered the diversity of the microbiota and altered the microbial community of the gut in *Strauchbufo radde* [14]. In Asian toads (*Bufo gargarizans*), life cycle stage is the main factor driving differences in gut microbiota between populations [15]. In green frog tadpoles (*Lithobates clamitans*), both ambient temperature and microbial communities affect relative brain mass and shape [16]. As a metameric vertebrate, frog larvae (tadpoles) live in water, while most adults live on land, and they have characteristics intermediate between those of fish and reptiles. This lifestyle also creates its unique gut microbiota. It has been noted that the gut microflora of tadpoles is similar to that of fish [17], while adult frogs are more like mammals than fish [18]. This is determined by their lifestyle and environment. The sex differentiation of amphibians occurs during the development of larva [19]. The baseline corticosterone release rate of amphibians is positively correlated with bacterial diversity [20]. At the same time, corticosterone inhibits plasma androgen concentration by inhibiting the release of LH-RH from the hypothalamus, thus affecting its sex differentiation [21]. Furthermore, a study on mice showed that the external light–dark cycle shapes the gut microbiota through retinal ganglion cells that are essentially light-sensitive [22]. Therefore, the intestinal flora of the tadpole stage is worthy of attention.

The Dybowski’s frog (*Rana dybowskii*) is a widely distributed amphibian found in northeastern regions. The species holds high ecological value within forest ecosystems and even greater economic importance due to its fallopian tubes, which are traditionally processed into a valuable medicinal product known as Oviductus Ranae, listed in the *Chinese Pharmacopoeia* (2020 edition) [23]. Modern pharmacological studies have confirmed its immune-regulatory and anti-glioma properties, enhancing its market demand [13,24]. Notably, *R. dybowskii* displays strong sex-based economic differentiation, with females being far more valuable due to their reproductive organs and eggs. As a result, understanding and regulating sex differentiation during early developmental stages has become a key focus for both researchers and industry. The tadpole stage of *R. dybowskii* is critical for metamorphosis and sex differentiation, yet it remains poorly understood under artificial conditions [25]. Given that the light spectrum is a cost-effective and manipulable factor in aquaculture, this study aims to explore the effects of different light colors on tadpole growth, steroid hormone fluctuations, and gut microbiota composition. The findings offer valuable insights for improving sex ratio control and overall rearing efficiency in artificial breeding while also laying a foundation for future field-based spectral ecology studies.

## 2. Materials and Methods

### 2.1. Sample Collection

Fertilized eggs of *Rana dybowskii* were obtained from a commercial frog farm in Dandong, Liaoning Province, China, and transported to the aquaculture laboratory of Shenyang Agricultural University on April 5th. The eggs were incubated in a recirculating water system under room temperature (11.93 ± 3.59 °C) and light conditions (12L:12D). After seven days of incubation, the hatching of the tadpole egg mass were successfully completed, and 1500 healthy tadpoles in stage 25 (0.06 ± 0.01 g) were randomly assigned to 15 culture boxes (50 × 35 × 20 cm, 100 tadpoles per box). These were divided into five light treatment groups, white (N), red (R), yellow (Y), blue (B), and green (G), with three replicates per group. Culture boxes were placed on racks with external blackout curtains, and light bulbs were centrally positioned to provide a uniform intensity (571 ± 184.13 Lx) at the water surface. The water depth was maintained at 6 cm, and a 12L:12D photoperiod was applied. Meanwhile, the tadpoles were fed twice a day. When changing the water, we checked the status of the tadpoles and removed the dead individuals in time.

Tadpoles were reared on an artificial diet (Wellhope Frog Complementary Pellet Feed, Shenyang, Liaoning Province, China) for 60 days, with measurements of body weight, metamorphosis rate, and survival rate recorded every 15 days. Additionally, at each 15-day interval, 15 tadpoles were randomly selected from each light treatment group, and their viscera were dissected and pooled into three sterile 2 mL centrifuge tubes (five individuals per tube). These samples were stored at –80 °C for subsequent high-performance liquid chromatography (HPLC) analysis. After the 60-day experiment, a total of 60 metamorphosed juveniles were randomly collected from three replicates per light treatment (20 per replicate); of these, 36 individuals were used for sex ratio analysis and 24 for gut microbiota analysis. The juveniles were anesthetized with 800 mg/L MS-222 solution for 3 min, and intestinal tissues were immediately extracted. We removed the non-research content from the intestinal tissues, cleaned the tissue stains with 1×PBS prepared with pre-cooled nuclease-free water at 2-6 °C or normal saline, and aspirated the liquid on the surface. Tadpole internal organ samples were flash-frozen in liquid nitrogen, pulverized in a grinding bowl, and subjected to total RNA isolation using Trizol Reagent (RNAiso PLUS; Takara, Dalian, China). RNA integrity was verified by 1% agarose gel electrophoresis, while purity and concentration were determined using a NanoDrop spectrophotometer (Thermo Scientific, Shanghai, China).

### 2.2. Basic Metrics of R. dybowskii

At the end of the experiment, the average body weight (AW), survival rate (SR), metamorphosis rate (MR), and female rate (FR) of tadpoles were analyzed, and the calculation formula was as follows: AW (g) = W/N, where W is the total weight of incomplete and complete metamorphosed tadpoles (g) and N is the number of incomplete or completely metamorphosed tadpoles; SR (%) = Nt/Np × 100%, where Nt is the total number of surviving tadpoles and Np is the initial survival number and MR (%) = Nm/N_R_, where Nm refers to the total number of tadpoles that have developed forelimbs [26], while N_R_ refers to the number of tadpoles in each group.

### 2.3. Sex Identification Method Based on Gene Expression

In the two species of frogs, *Nanorana quadranus* and *Quasipaa yei*, *Dmrt1* is considered to be the sex-determining gene [27]. In the Dybowski’s frog, *Cyp19a1*, *Foxl2*, *Nr5a1*, *Wnt*, and *Hsd* are considered to be involved in the sex determination of females. Therefore, the sex-determining genes of *R. dybowskii* were identified by using RNA sequencing (RNA-seq) gonadal tissues from adult males and females in our previous study [28]. Differentially expressed genes related to sex differentiation, including *Cyp19a1*, *Cyp17a1*, *Sox9*, *Sox3*, *Dmrt1*, *Fem 1b*, *Foxl2*, and *Amh*, were screened. To validate the RNA-seq results and evaluate their applicability to juvenile frogs, fluorescence quantitative PCR (qPCR) was performed on both adult and juvenile gonadal samples (Table S1). Among the candidate genes, *Cyp19a1* and *Foxl2* exhibited consistent sex-specific expression patterns in both developmental stages. The amplification efficiencies of each gene are shown in the attached table (Table S2).

We used this method to identify 36 metamorphosed juvenile frogs in different light color treatment groups to calculate the female rate. The 20 µL reaction mixture contained 2 µL cDNA, 0.4 µL SupRealQ Purple Universal SYBR qPCR Master Mix (U+) (Vazyme, Nanjing, China) for each primer, and 7.2 µL RNase-free water. Cycling conditions comprised the following: pre-denaturation at 50 °C for 120 s; denaturation at 95 °C for 120 s; 40 cycles of amplification (95 °C for 15 s, 60 °C for 15 s, 72 °C for 30 s); and a melting curve analysis (95 °C for 15 s, 60 °C for 60 s, 95 °C for 15 s). The melting curve analysis was conducted using LC96 software (version SW1.1), and the relative expression levels of the target gene were calculated using the 2^−ΔΔ^Ct method.

### 2.4. Hormone Quantification via HPLC

Four hormones—cortisol (COR), testosterone (T), estradiol (E2), and androstenedione (A)—were quantified by HPLC method (Dionex Ultimate 3000, Thermo Scientific, Waltham, MA, USA). Hormone standards, including androstenedione (A), estradiol (E2), testosterone (T), cortisol (COR), and progesterone (P), were procured from Beijing Solarbio Science & Technology Co., Ltd. (Beijing, China). Chromatographic-grade methanol was obtained from FUYU CHEMICAL. Methanol and ultrapure water were individually filtered through 0.45 µm and 0.22 µm membranes, respectively, mixed in a 1:1 ratio, degassed by sonication for 15 min, and stored in the dark. Hormone standards were dissolved in the prepared mobile phase, stored at −2 °C, diluted with the mobile phase to achieve working solutions ranging from 0.1 to 5000 ng/g, and filtered through a 0.22 µm membrane prior to use.

We took the tadpoles that were quick-frozen in liquid nitrogen and placed them on qualitative filter paper. Ice cubes were laid under the filter paper. We removed the internal organ masses, ground them evenly, and then added an appropriate amount of chromatograph-grade methanol solution. We placed the mixture in a 4 °C refrigerator for cold immersion for 4 h. After this, centrifugation was carried out using a low-temperature centrifuge, and then the supernatant was taken out. The remaining residue was extracted for the second time to ensure that the hormone was fully extracted. The steps were the same as the first time. Finally, we mixed the supernatants extracted from the two times. The supernatants were combined and diluted with an equal volume of ultrapure water. The resulting solution was filtered through a 0.22 µm membrane to prepare the high-performance liquid chromatography (HPLC) test solution. Chromatographic separation was performed on a Thermo HPLC system equipped with a 50 × 2.1 mm (1.8 µm) column at 30 °C, using a detection wavelength of 230 nm and a mobile phase consisting of methanol (A) and ultrapure water (B) under gradient elution at a flow rate of 0.2 mL/min. Standard mixtures were injected every nine samples to ensure system stability. Standard curves for T, A, E2, COR, and P were constructed, achieving R^2^ values between 0.9989 and 0.9995 (Table S3).

### 2.5. DNA Extraction and 16S rRNA Gene Sequencing

Total microbial DNA was extracted using the Tengen Bacterial Genome Extraction Kit (DP302-02) and quantified with a Qubit fluorometer (Invitrogen, Waltham, MA, USA). The V3-V4 region of the 16S rRNA gene was amplified via PCR using primers 341F and 805R under the following conditions: 98 °C for 30 s (pre-denaturation), 32 cycles of 98 °C for 10 s (denaturation), 54 °C for 30 s (annealing), and 72 °C for 45 s (extension), followed by a final extension at 72 °C for 10 min. PCR products were purified with AMPure XP beads (Beckman Coulter, Brea, CA, USA) and quantified using Qubit and an Agilent 2100 Bioanalyzer (Agilent, Frederick, CO, USA). Qualified libraries (≥2 nM) were pooled, denatured with NaOH, and sequenced on the NovaSeq 6000 platform (2 × 250 bp) using a NovaSeq 6000 SP Reagent Kit (500 cycles).

### 2.6. Bioinformatics

Microbiome bioinformatics analysis was performed using QIIME2 (version 2019.4), following the official tutorial (https://docs.qiime2.org/2019.4/tutorials/; accessed on 30 August 2024) with minor modifications. Raw sequencing data were initially processed by demultiplexing with the q2-demux plugin followed by primer removal using the q2-cutadapt plugin [29]. Sequencing reads were processed using the DADA2 plugin for quality filtering, noise reduction, read merging, and chimera removal [30]. Bioinformatics analysis was conducted using QIIME2 and R package v3.2.0. At the ASV level, alpha diversity indices (Chao1, observed species, Shannon, Simpson) were derived from QIIME2-generated ASV tables and presented in box plots. Abundance curves were employed to compare ASV distribution across samples. Beta diversity analysis quantifying microbial community differences utilized Bray–Curtis dissimilarity, visualized via principal coordinate analysis (PCoA), non-metric multidimensional scaling (NMDS), and hierarchical clustering [31]. A Venn diagram was constructed using the R package VennDiagram to visualize shared and unique ASVs across sample groups, based exclusively on ASV presence/absence data irrespective of relative abundance [32].

### 2.7. Data Analysis

All statistical analyses were conducted using SPSS 27.0. Growth, survival, and metamorphosis data were analyzed via one-way ANOVA followed by Duncan’s multiple range test (*p* < 0.05). HPLC detection conditions were the same as for the standards. The peak values of each hormone in the tadpole visceral mass samples were measured, and the concentration of each test sample was calculated using the linear regression equation of each standard. The significance level of differences for all analyses was set at *p*-value < 0.05, and data are presented as mean ± standard error. Microbial diversity indices were compared using the Kruskal–Wallis test (*p* < 0.05).

## 3. Results

### 3.1. Growth, Survival, Metamorphosis, and Sex Ratio

**Figure 1:**
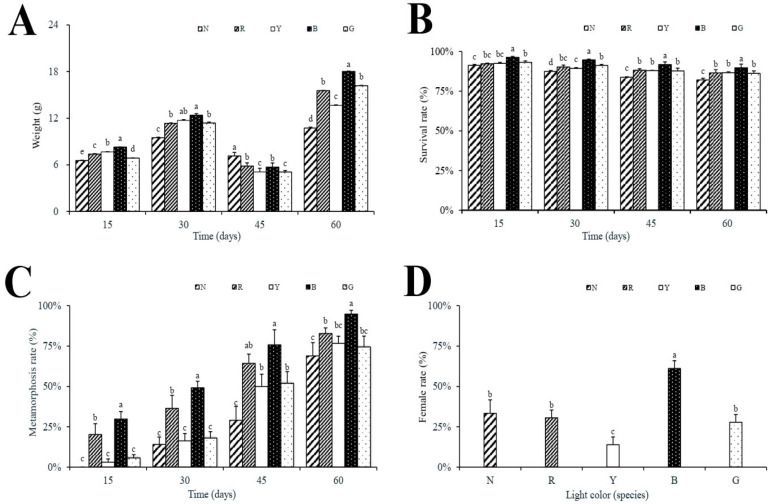
Comprehensive growth parameters at different time points. (**A**) Body weight, (**B**) survival rate, (**C**) metamorphosis rate, (**D**) female rate. Description of all panels: Data are mean ± SEM (*n* = 3 tank replicates per group; panels (**A**–**C**): 14 tadpoles per tank, panel (**D**): 11 tadpoles per tank). Significant differences determined by one-way ANOVA with Tukey’s post hoc test: different letters show significant differences *p* < 0.05.

The peak weight appeared on day 60, which was 10.74 ± 0.57 g (N), 15.55 ± 0.35 g (R), 13.64 ± 0.39 g (Y), 18.01 ± 0.86 g (B), and 16.18 ± 0.60 g (G), respectively (Figure 1A). Group B was significantly higher than other treatment groups. Moreover, the survival rate of group B was significantly higher than that of other groups and reached 89.72 ± 2.37% on day 60 (Figure 1B). The metamorphosis rate of group B was significantly higher than that of other treatment groups on days 15, 30, and 60, which was 29.82 ± 4.62%, 49.12 ± 4.12%, and 94.79 ± 2.39%, respectively (Figure 1C). Furthermore, the female rate in group B was significantly higher than that in the other groups, reaching 61.11% (Figure 1D).

### 3.2. Steroid Hormone Levels

**Figure 2:**
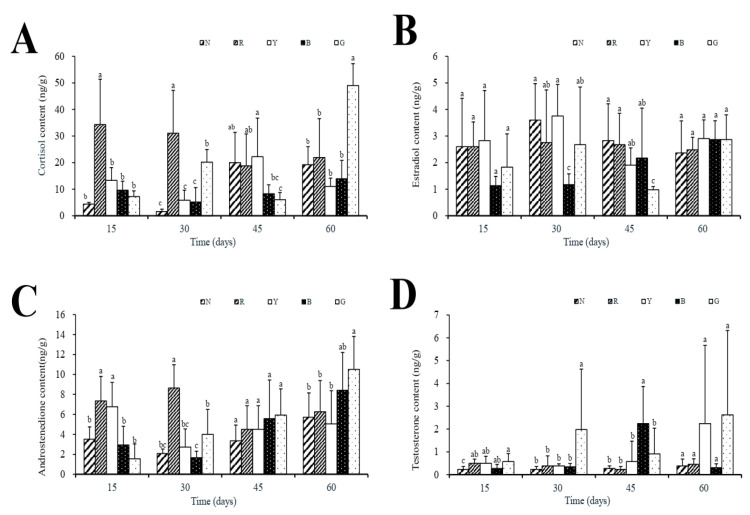
Steroid hormone levels at different time points. (**A**) Cortisol, (**B**) estradiol, (**C**) androstenedione, (**D**) testosterone. Statistical comparisons were performed between treatment groups at each time point; temporal changes within each group were not analyzed as this was not this study’s primary focus. Data expressed as mean ± SEM (*n* = 3 replicates). Significant differences determined by one-way ANOVA with Tukey’s post hoc test: different letters show significant differences *p* < 0.05.

Cortisol content in group R reached its peak on day 15 (34.33 ± 17.06 ng/g) and decreased to 31.04 ± 16.17 ng/g on day 30. However, in the G group, cortisol levels peaked at 49.01 ± 8.24 ng/g on day 60. The cortisol content of group B was low and stable, reaching a peak of 13.94 ± 6.70 ng/g on the 60th day (Figure 2A). The estradiol content of group B increased with the increase in time and reached its peak value (2.87 ± 0.71 ng/g) on the 60th day (Figure 2B). Meanwhile, the content of androstenedione in group G also increased with the increase in time, reaching 10.51 ± 3.30 ng/g on the 60th day (Figure 2C). The contents of testosterone in groups Y and G both peaked on day 60, reaching 2.24 ± 3.43 ng/g and 2.62 ± 3.70 ng/g, respectively (Figure 2D).

### 3.3. Pyrosequencing of the Gut Bacterial Community

The intestinal samples yielded a total of 127,1183 raw sequence reads, with an average of 84,746 reads per sample. Following the implementation of quality filtering and denoising procedures, 113,8817 clean reads remained, with an average of 75,951 reads per sample. A total of 2056 ASVs were identified at various taxonomic levels (Table S4). Statistical analysis of intestinal ASVs demonstrated that the number of independent ASVs in groups N, R, Y, B, and G were 180, 167, 417, 295, and 147, respectively, and the number of ASVs shared by the five groups was 168, accounting for 8.17% of the total number of ASVs (Figure S1).

### 3.4. Microbial Alpha Diversity

**Figure 3:**
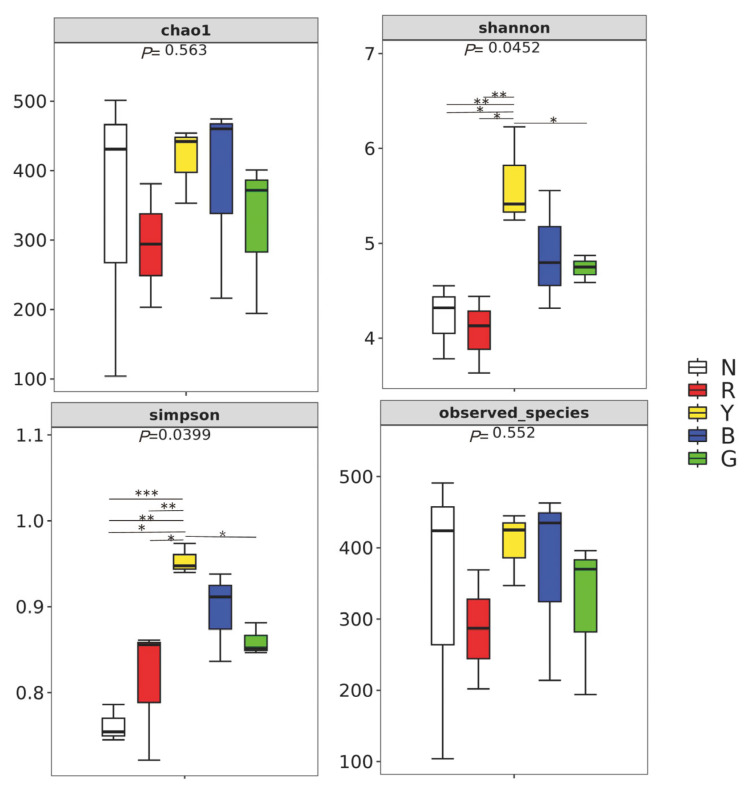
The alpha diversity index of intestinal bacterial communities. Box plots depict the medians (central horizontal lines), inter-quartile ranges (boxes), and 95% confidence intervals (whiskers). *p*-value are from Kruskal–Wallis test. Asterisks indicate statistically significant differences between pairs of values (* *p* < 0.05, ** *p* < 0.01, and *** *p* < 0.001).

The assessment of bacterial abundance and diversity was conducted in five distinct light colors: white light, red light, yellow light, blue light, and green light, utilizing the alpha diversity index (Figure 3). The Chao1 index and Observed_species index exhibited no significant disparities among the light groups. The Shannon index under yellow light was not significantly different from group B, but it was significantly different from other groups. At the same time, the Simpson index under yellow light was not significantly different from group B, but it was significantly different from other groups. This finding suggests that the intestinal bacterial community richness and diversity of *R. dybowskii* larvae is maximized under yellow light, followed by blue light.

### 3.5. Gut Bacterial Taxonomic Abundance

**Figure 4:**
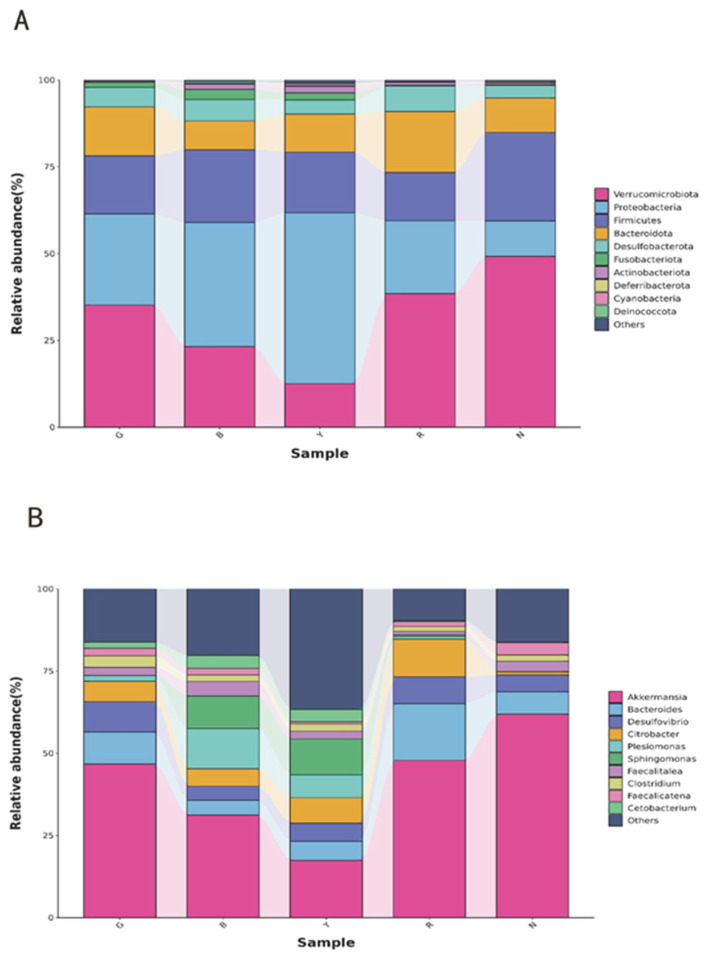
Relative abundance of intestinal bacterial communities: (**A**) phylum level, (**B**) genus level.

The relative abundance of the gut flora of *R. dybowskii* at the phylum level was analyzed by sequence comparison and annotation (Figure 4A). The predominant phyla were *Verrucomicrobia*, *Proteobacteria*, *Firmicutes*, and *Bacteroidetes*, accounting for over 85% of the total relative abundance of the gut microbiota. However, the proportions vary with different light colors. The most prevalent population of *Verrucomicrobia* was observed under white light, with a percentage yield of 48.26%. This was followed by red light, which yielded 37.97%, and green light, with a percentage yield of 34.97%. In contrast, under blue light and yellow light, the predominant bacterial population was *Proteobacteria*, with percentages of 35.58% and 48.70%, respectively.

As illustrated in Figure 4B, the most prevalent genus of *R. dybowskii* gut flora at the genus level was *Mucinophilus-Akkermansiamuciniphila*. However, the relative abundance of other genera varied under different light colors. *Sphingomonas* demonstrated the highest relative abundance under blue and yellow light, the lowest in the other groups, and an absence of *Sphingomonas* under green light. *Citrobacter* demonstrated a relative abundance of less than 1% under white light, while in the other groups, the relative abundance was higher, with the highest recorded in group R at 9.08%. Overall, the light color altered the structure of the flora at the level of microbial genera in the gut of *R. dybowskii*.

### 3.6. Beta Diversity

The beta diversity index can reflect changes in the structure of the intestinal bacterial community, and we obtained the beta diversity index (Bray–Curtis distance) using PCoA and NMDS analyses (Figure S2) The results showed that there was a partial overlap between the white light group and the red and blue light groups, indicating that there was no significant difference in species composition of the intestinal flora under white light, red light, and blue light. In contrast, yellow light, green light, and white light had no overlap, indicating that there was a significant difference in the species composition of the intestinal flora under yellow light, green light, and white light.

### 3.7. Taxonomic Differences and Marker Species

**Figure 5:**
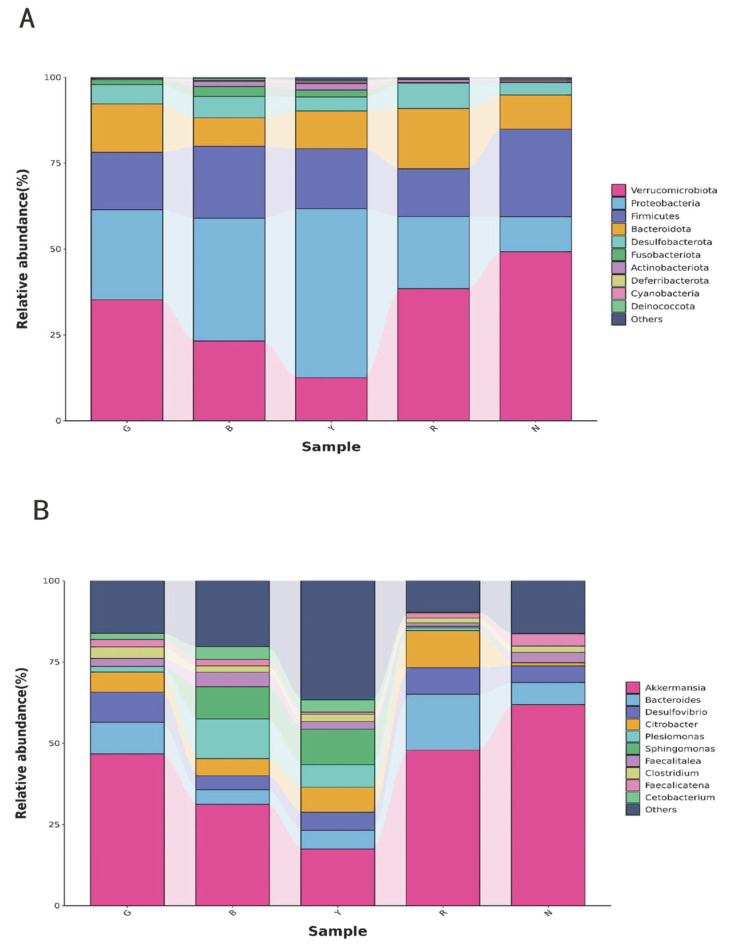
Correlation analysis of intestinal bacteria: (**A**) phylum level, (**B**) genus level.

According to the rich heat map at the gate level (Figure 5A), the intestinal bacterial community structure changed significantly under different light colors. *Cyanobacteria*, *Chloroflexi*, *Campylobacterota*, *NB1-j*, and *Bdellovibrionota* were only present in group Y. *Verrucomicrobiota* was the least abundant in group Y. According to the abundance heat map at the genus level (Figure 5B), among the 20 genera, *Anaerorhabdus_furcosa_group* and *Methylobacteria-methylorubrum* had high abundance only in group Y. *Clostridium*, *Alistipes*, and *Aeromonas* had high abundance only in group G, *Plesiomonas* had high abundance only in group B, and *Bacteroides* and *Citrobacter* had high abundance only in group R. This further proves that the phylum and genus abundance of the intestinal flora is affected by the change in light color.

## 4. Discussion

The present results showed that on day 15, survival rate under blue light was significantly higher than all treatment groups, while survival under red light was lower. This finding is consistent with previous studies reporting that red light negatively affects the survival of early developmental stages in several amphibian species, including *Triturus cristatus*, *Rana arvalis*, and *Rana temporaria* [33]. At the same time, our study found that the average body weight of tadpoles exposed to blue light was significantly higher than that of tadpoles exposed to red light at days 15, 30, and 60. Blue light was also found to accelerate growth and development in tadpoles (*Polypedates teraiensis*), while red light slowed growth [34]. The metamorphosis development time of American toads (*Anaxyrus americanus*) treated with blue artificial light at night was reduced by about 30% in the larval stage [35]. In addition, blue light can also shorten the metamorphosis time of *Pelophylax ridibundus* [3]. This was confirmed by our results, which showed that, overall, the metamorphosis rate under blue light was significantly higher than in the treatment groups.

In amphibians, the hypothalamic–pituitary–gonadal axis (HPG) is a key endocrine system controlling gonad development and sex hormone synthesis [36]. This axis directly innervates the anterior pituitary through gonadotropin-releasing hormone (GnRH) neurons, releasing GnRH, which stimulates the synthesis and release of luteinizing hormone (LH) and follicle-stimulating hormone (FSH). These gonadotropins act on the gonads and other organs through the blood circulation, thereby regulating the secretion and release of sex hormones [37]. Measurement of steroid hormone levels in tadpoles can directly reflect the gonad development status in the body, and sex hormones play a crucial role in the process of amphibian sex differentiation [38]. Our study found that tadpoles exposed to green light had higher levels of testosterone on days 15, 30, and 60. This is similar to the findings of a fish study. A study of *Scomber japonicus* showed that green light significantly increased plasma testosterone levels in fish [39]. At the same time, the continuous increase in estradiol content under blue light may be related to the regulation of the hypothalamic–pituitary–gonadal axis (HPG axis) by light color. A previous mouse study reported that exposure to blue light for 6 h increased estradiol concentrations in female rats [40]. These findings reveal the important role of light color in the endocrine regulation of tadpoles.

Endogenous estradiol (E2) plays a stimulating role in female development of tadpoles by regulating gene expression of estrogen and androgen receptors, while endogenous testosterone (T) in tadpoles can lead to masculinity [19]. Therefore, we infer that blue light enhances the feminization of *R. dybowskii* tadpoles, while green light promotes their masculinization. Our results on female rates also confirm this hypothesis. In addition, cortisol is a marker of stress [41]. In this study, we found that in the first 30 days, the cortisol content of the light group was higher than that of the natural group (group N), especially the R group, where the cortisol content was very high. This indicates that the light color caused considerable stress to the northeast forest frog. In a study by Forsburg et al., artificial light at night disrupted the light–dark cycle in the natural environment and disrupted circadian rhythms, which can alter glucocorticoid levels in amphibians [42]. One point of concern is that we found that the red light also shows a premature metamorphosis of young frogs, and we concluded that this may be because tadpoles produced greater stress to red light and had higher cortisol content in the body, which affected the start time of their metamorphosis. Studies on *Xenopus laevis* report that cortisol regulates the initiation and overall rate of metamorphosis development by affecting plasma hormone levels [43]. However, prolonged interference can lead to a sustained rise or fall in glucocorticoids, which can be harmful [44]. In Hylarana indica, cortisol causes a slower rate of growth and development, especially in the early and middle stages of larval development [45]. Therefore, we believe that blue light not only promotes the growth, development, and metamorphosis of *R. dybowskii* tadpoles but also enhances their female ratio.

The gut microbiota is essential for maintaining the internal homeostasis of the host and plays an important role in growth and development [46]. In the natural environment, gut microbes are influenced by environmental factors and the host itself, and there are some differences in their structure and composition [47]. In aquatic organisms, there are significant differences in the composition of gut microbial communities. For example, the gut microbial community of fish is rich in *Proteobacteria* [48], whereas amphibians are dominated by *Firmicutes* and *Bacteroidetes* [49]. The core flora of the *R. dybowskii* gut at the phylum level in this study comprised *Verrucomicrobia*, *Proteobacteria*, *Firmicutes*, and *Bacteroidetes*; this is similar to the abundance of gut flora found in some frogs such as *Babina adenopleura* [50], *Polypedates megacephalus* [51], and *Odorrana tormota* [52], suggesting that *Proteobacteria*, *Firmicutes*, and *Bacteroidetes* are the dominant phyla in the amphibian gut microbiota. However, in the present study, the abundance of *Verrucomicrobia* accounted for a relatively high percentage, which is very rare, and the relative abundance of *Verrucomicrobia* varied among amphibian larvae from different habitats, which may be related to the type of food they ingested and habitat conditions [53]. *Akkermansia muciniphila* is one of the representative bacteria of *Verrucomicrobia*, which accounted for a relatively high abundance in this study. It was found that mice were able to significantly increase the abundance of *Akkermansia muciniphila* in the gastrointestinal tract after consuming the antioxidant-rich tropical fruit Camu Camu [54]. Mice fed fish oil alone also had a significant increase in the abundance of *Akkermansia muciniphila* in their intestines [55]. In the present study, *R. dybowskii* tadpoles were fed only yellow mealworms, which are rich in unsaturated fatty acids and antioxidants [56], after complete metamorphosis. This may have contributed to the increased abundance of *Akkermansia muciniphila* in the present study. This is also a drawback of this study, as studies have shown that captive amphibians have lower bacterial species diversity and a lower relative abundance of microbiota compared to wild individuals, which may lead to greater susceptibility to infection [57]. Furthermore, whether in captivity or in the wild, *R. dybowskii* should be significantly enriched in *Bacteroidetes* and *Firmicutes*, which is different from the significant increase in the abundance of *Akkermansia muciniphila* in this study [58].

In this study, the alpha diversity analysis of intestinal microorganisms of *R. dybowskii* found that there were significant differences in Shannon index and Simpson index between group Y and group N and between Group R and group G but no significant differences between group B and group Y. At the same time, the results of PcoA and NMDS showed that the species composition of intestinal flora under yellow light, green light, and white light was significantly different. These results showed that the intestinal bacterial community richness and diversity of tadpoles were highest in yellow light, followed by blue light. These findings reveal the important role of light color in the regulation of gut microbes in tadpoles of *R. dybowskii*. Studies have shown that exposure to blue light can increase the richness and diversity of gut bacteria in ducks [59]. In addition, a study in chickens showed that blue and blue–green complex light can also affect the composition of their gut microbiota [60]. Studies on yellow light and intestinal flora have not been reported. We therefore believe that blue light can effectively increase the richness and diversity of intestinal bacteria in tadpoles.

There were differences in the classification and abundance of intestinal bacteria in different light colors, and according to the gate-level heat map, only *Bdellovibrionota* had a higher abundance in group Y. *Bdellovibrionota* are a special group of bacteria that feed on other bacteria and have the ability to metabolize osmoprotectants and degrade the cell wall [61]. In contrast, the abundance of *Verrucomicrobia* was decreased in group Y, which may be related to the decreased ability of lipid metabolism. Thus, in the amphibian gut, the metabolic capacity of *Bdellovibrionota* may contribute to host digestion and absorption of nutrients. Genus-level heat maps showed that *Plesiomonas* was only highly abundant in group B. A study in zebrafish showed that the relative abundance of *Plesiomonas* was positively correlated with the expression of immune-related genes [62]. And a study in mice showed that immune metabolism plays a bimodal role at the interface of extracellular immune response and intracellular metabolism, controlling intracellular processes and extracellular inflammatory responses by regulating cellular energy supply and demand [63]. Based on this evidence, we hypothesize that different light treatments affected the stress levels of tadpoles, and only the tadpoles in group B made rapid adjustments. Therefore, although the intestinal flora richness and diversity of tadpoles were greater under yellow light, their lipid metabolism may be reduced and their anti-stress level may not be as good as under blue light. Considering growth and development, hormone levels, etc., we agreed that blue light is more suitable for *R. dybowskii* tadpoles.

## 5. Conclusions

Different light colors can effectively affect the growth and development, hormone levels, and intestinal flora composition of *R. dybowskii* tadpoles. Under blue light, *R. dybowskii* tadpoles had a higher metamorphosis rate, average body weight, female rate, and survival rate. Red light tended to stress the tadpoles, while blue light was able to increase estradiol levels in the tadpoles, possibly increasing the proportion of *R. dybowskii* females. Yellow light, while having higher gut bacterial abundance and diversity, may also be associated with decreased lipid metabolic capacity and anti-stress ability. Therefore, blue light is the most suitable light color for the growth and development of *R. dybowskii* tadpoles. The present study has some limitations, such as feeding tadpoles only with yellow mealworms after complete metamorphosis, which affected the composition of their gut flora to some extent. In conclusion, our results contribute to the understanding of the effects of light coloration on *R. dybowskii* tadpoles and provide important insights for improving their rearing conditions. Meanwhile, our research results also provide basic data for conducting spectral experiments on *R. dybowskii* in the field.

## Data Availability

The raw reads have been deposited into NCBI database (BioSample number SAMN48144476).

## Supplementary Materials

The following supporting information can be downloaded at: https://www.mdpi.com/article/10.3390/ani15142066/s1, Figure S1: Venn; Figure S2: PCoA and NMDS analysis; Figure S3: Cumulative peak chromatogram; Table S1: The information of genes validated by qRT-PCR; Table S2: Data on gene amplification efficiency; Table S3: Standard curve; Table S4: Sequencing data of intestinal flora of *Rana dybowskii*.
