# Supplementary figures and images for: Impact of Light Spectrum on Tadpole Physiology and Gut Microbiota in the Dybowski’s Frog (Rana dybowskii)
Source: Animals (Basel). 2025 Jul 13;15(14):2066. doi: 10.3390/ani15142066 (PMC12291657; doi:10.3390/ani15142066)

# Venn Diagram

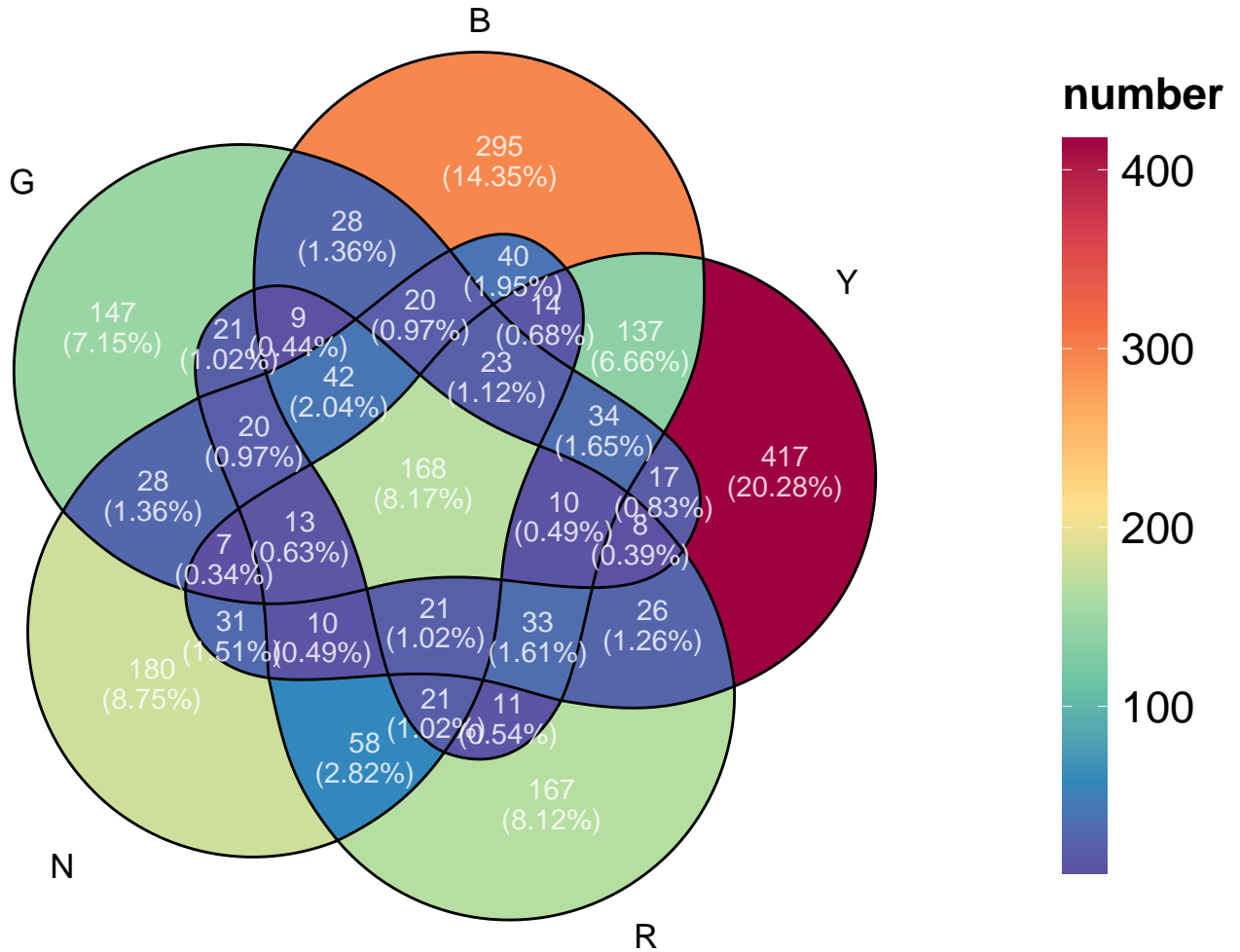

Supplement: Supplementary file 1 [file animals-15-02066-s001.zip › Figure S1. Venn.pdf]

A

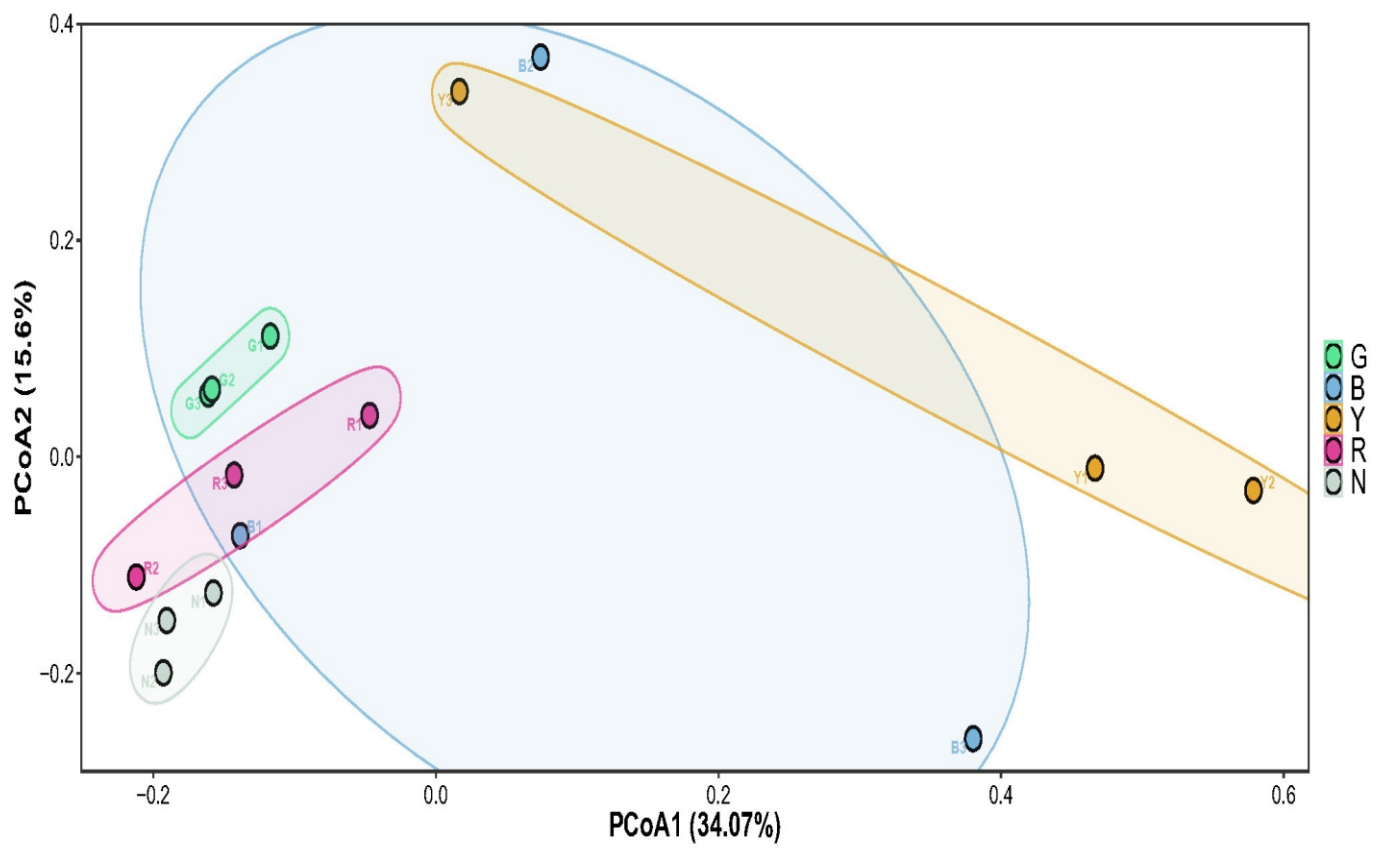

B

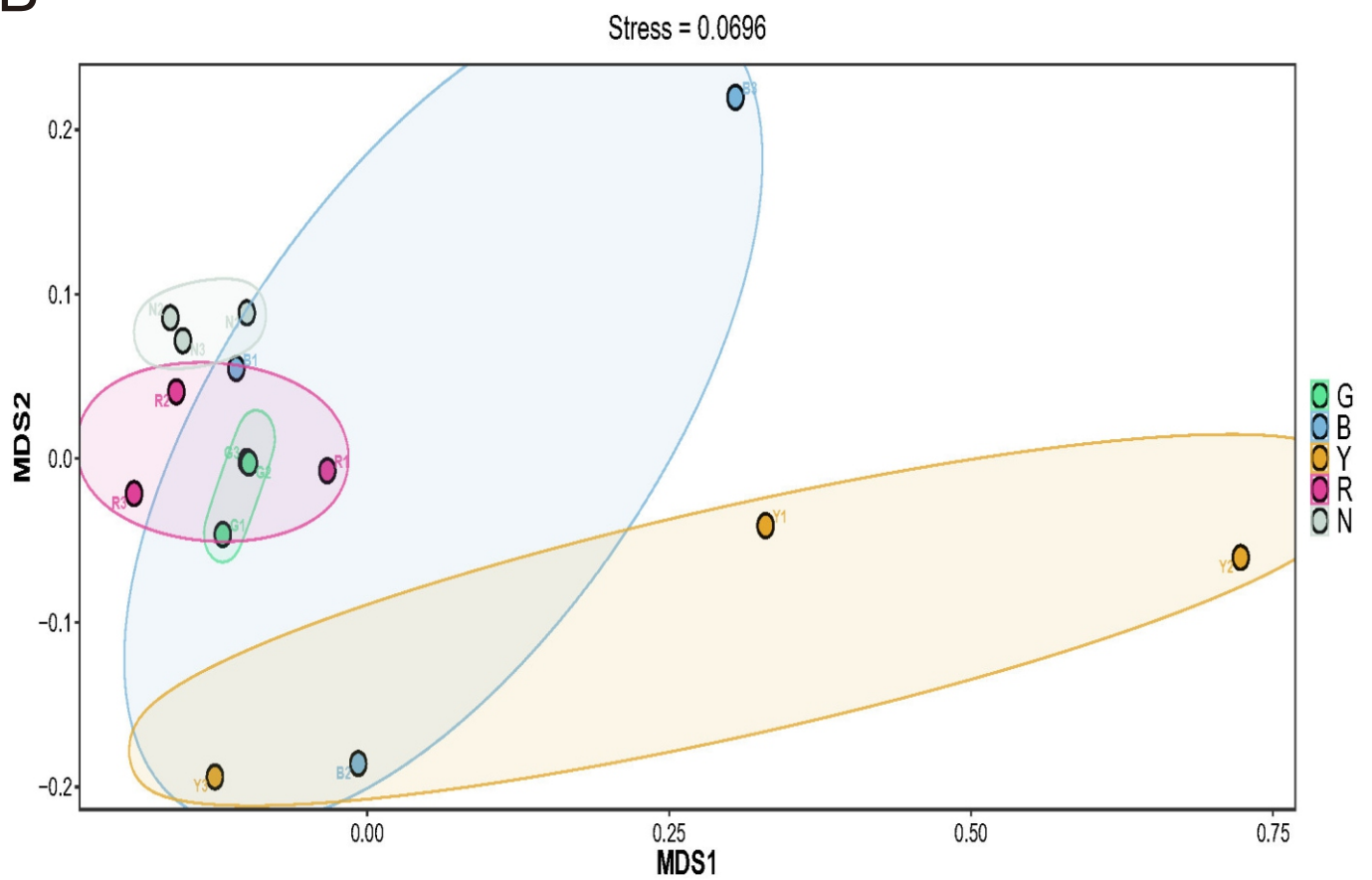

Supplement: Supplementary file 1 [file animals-15-02066-s001.zip › Figure S2. PCoA and NMDS analysis.pdf]

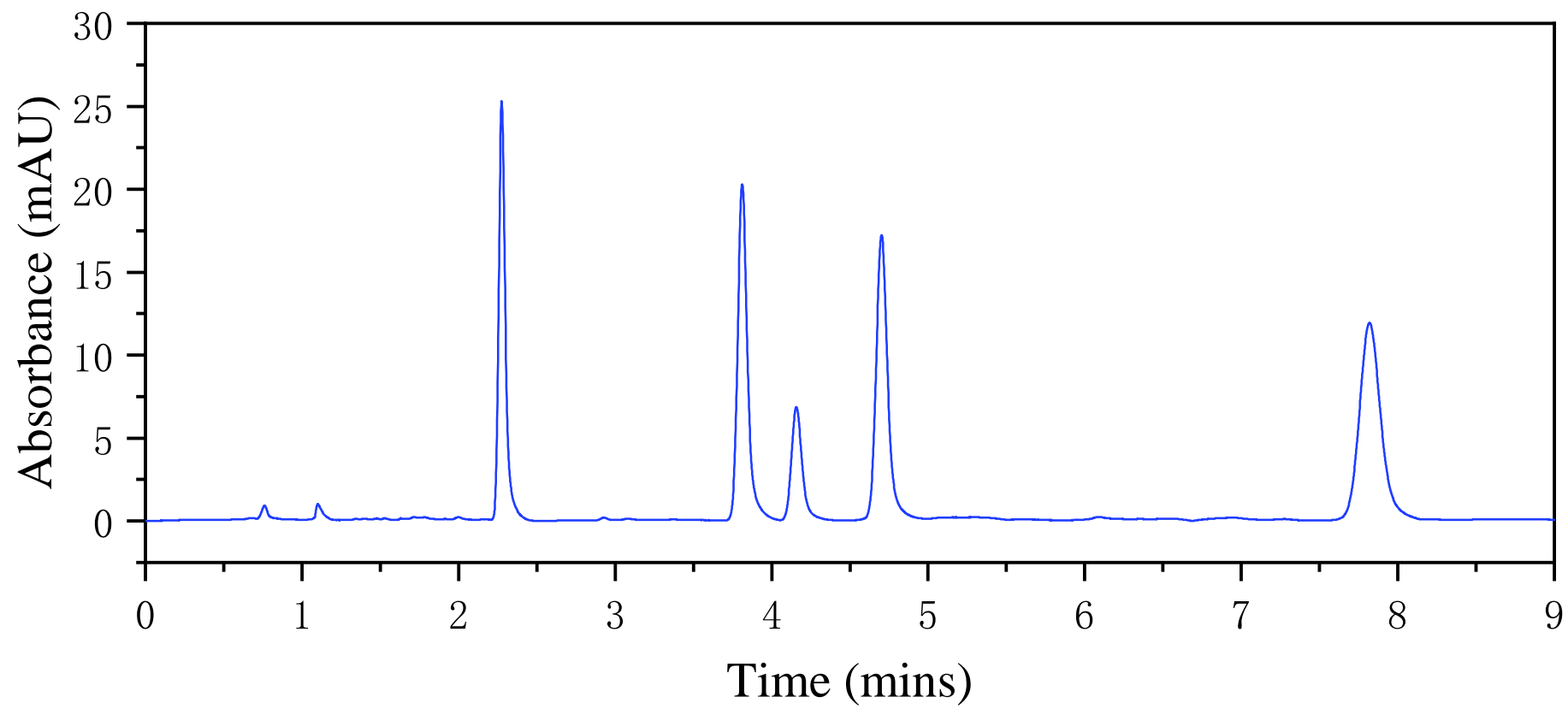

Supplement: Supplementary file 1 [file animals-15-02066-s001.zip › Figure S3. Cumulative peak chromatogram.pdf]
